# Four new species of *Resupinatus* (*Agaricales*, *Basidiomycota*) on coniferous trees in Northeast China

**DOI:** 10.3897/mycokeys.132.188107

**Published:** 2026-05-15

**Authors:** Lei Zhuang, Qian-Xin Guan, Yu-Cheng Dai, Yuan Yuan, Heng Zhao

**Affiliations:** 1 School of Ecology and Nature Conservation, Beijing Forestry University, Beijing 100083, China School of Ecology and Nature Conservation, Beijing Forestry University Beijing China; 2 CAS Key Laboratory of Forest Ecology and Silviculture, Institute of Applied Ecology, Chinese Academy of Sciences, Shenyang 110164, China Harbin Academy of Agricultural Sciences Harbin China; 3 Harbin Academy of Agricultural Sciences, Harbin 150029, Heilongjiang, China Institute of Applied Ecology, Chinese Academy of Sciences Shenyang China

**Keywords:** East Asia, fungal diversity, new taxa, temperate-boreal, wood-inhabiting fungi

## Abstract

*Resupinatus* species are widely distributed worldwide and are characterized by pleurotoid, cupulate-lamellate, cyphelloid, poroid, and merulioid basidiomata forms. Although numerous species have been described from subtropical to tropical regions in recent years, the species diversity of *Resupinatus* in temperate-boreal coniferous forests remains poorly investigated. In this study, four new species are described on the basis of morphological observations and molecular phylogenetic analyses. *Resupinatus angulatus* is characterized by angular isolated pores, fusiform cystidioles, and curved cylindrical to allantoid basidiospores, and it occurs on fallen branches of *Larix olgensis* and *Pinus koraiensis*. *Resupinatus latemarginatus* is distinguished by circular to subcircular isolated pores, a wide sterile margin, fusiform cystidioles, ellipsoid basidiospores, and growth on fallen branches of *Abies nephrolepis* and *P. koraiensis*. *Resupinatus sinoapplicatus* is characterized by sessile basidiomata with a lamellate hymenophore, globose basidiospores, and growth on fallen branches of *P. koraiensis*. *Resupinatus sinuosus* is recognized by its sinuous to irregular isolated pores, fusiform cystidioles, and ellipsoid basidiospores, and it occurs on rotten wood of *A. nephrolepis*. This study expands knowledge of wood-decaying fungal diversity associated with temperate-boreal coniferous trees and highlights that fungal species diversity in this region remains insufficiently explored, warranting further investigation.

## Introduction

Wood-decaying fungi represent one of the most important groups of macrofungi, playing essential roles in energy flow and nutrient cycling within forest ecosystems (Dai et al. 2021; Dong et al. 2024). Meanwhile, they have substantial economic value, constituting a major source of edible and medicinal fungi (Wu et al. 2026). Traditionally, wood-decaying fungi include morphologically diverse groups such as polyporoid, corticioid, and hydnoid fungi, most of which are concentrated in the orders *Polyporales* and *Hymenochaetales* (Ryvarden et al. 2022; Hyde et al. 2024; Zhao et al. 2025a, 2026; Zhou et al. 2026; Zhu et al. 2026). In contrast, agaricoid fungi have received less attention (Zhang et al. 2023; Dong et al. 2025). Recently, with advances in mycological research, an increasing number of agaric fungi have been recognized as wood-inhabiting fungal species, particularly many genera within the order *Agaricales*, such as *Mycena* (Pers.) Roussel, *Marasmius* Fr., and *Resupinatus* Nees ex Gray (Dong et al. 2025).

The genus *Resupinatus* was proposed in 1821 and typified with *R. applicatus* (Batsch) Gray. *Resupinatus* is characterized by small, pleurotoid to cupulate-lamellate basidiomata, while cyphelloid, poroid, and merulioid forms are also included in the genus (Singer 1986; Redhead and Nagasawa 1987; Bodensteiner et al. 2004; Thorn et al. 2005; McDonald 2015; Bijeesh et al. 2020; Triantafyllou et al. 2026). Members of *Resupinatus* typically grow on decaying woody material and herbaceous debris (Gonou-Zagou et al. 2011; Carpouron et al. 2024; Liu et al. 2024). The genus has long been taxonomically problematic, as its morphology is easily confused with that of *Hohenbuehelia* species, which are characterized by a gelatinous layer in their pileus context and by colorless inamyloid spores (Thorn et al. 2005; McDonald 2015). The genus was historically placed in various families or treated as *incertae sedis* within *Agaricales* (Justo et al. 2011; He et al. 2024). Molecular phylogenetic studies later revealed a close relationship among *Resupinatus*, *Hohenbuehelia*, *Pleurotus* (Fr.) P. Kumm., and other related genera. Given multiple differences, including habit, pileipellis structure, and nematophagy, between *Pleurotaceae* (*Pleurotus* and *Hohenbuehelia*) and *Resupinataceae (Resupinatus)*, and the lack of significant support for their monophyletic origin, a monotypic family, *Resupinataceae*, was restored to accommodate *Resupinatus* within the suborder *Pleurotineae*, alongside *Fistulinaceae*, *Pleurotaceae*, and *Schizophyllaceae* (Vizzini et al. 2024). Accordingly, the genus *Resupinatus* belongs to *Resupinataceae*, *Agaricales*, *Agaricomycetes*, *Basidiomycota* (Hyde et al. 2024; Vizzini et al. 2024).

In recent years, many novel *Resupinatus* species have been reported on angiosperms, such as *R. odoratus* C.K. Pradeep, C. Bijeesh & A.M. Kumar, *R. tenuis* J.H. Dong & C.L. Zhao and *R. yunnanensis* Yang Yang & C.L. Zhao (Bijeesh et al. 2020; Yang et al. 2023; Dong et al. 2025). One species has been found on bamboos (*R. reviviscens* Carpouron & Raspé, in Carpouron et al. 2024), and two species have been found on coniferous wood (*R. abieticola* Triantafyllou & Gonou-Zagou, in Triantafyllou et al. 2026, and *R. porrigens* J.Z. Xu & Yu Li, in Liu et al. 2024). Previous studies have suggested that many wood-inhabiting fungi exhibit host tree preference (Purahong et al. 2018; Palla et al. 2023), implying that *Resupinatus* species may also be associated with specific host substrates. According to the MycoBank database (http://www.MycoBank.org, accessed on 2 April 2026) and Index Fungorum (http://www.indexfungorum.org, accessed on 2 April 2026), a total of 54 species of *Resupinatus* are currently accepted, including six known species described from China (Li and Bau 2014; Yang et al. 2023; Liu et al. 2024; Dong et al. 2025).

During investigations of macrofungi in Northeast China, several *Resupinatus* specimens were collected from coniferous wood, including *Abies nephrolepis* (Trautv. ex Maxim.) Maxim., *Larix olgensis* Henry, and *Pinus koraiensis* Siebold & Zucc. Based on the morphological and phylogenetic evidence, these specimens were identified as four novel species.

## Materials and methods

### Specimen collection

During the sampling trips, a total of 14 specimens were collected from Heilongjiang and Jilin provinces in Northeast China from August to October 2025. All specimens were deposited in the herbarium of the Institute of Microbiology, Beijing Forestry University (**BJFC**, China).

### Morphological observation

Morphological characterization was performed based on field observations and examination of dried specimens, following earlier taxonomic studies (Thorn et al. 2005; Carpouron et al. 2024; Dong et al. 2025). Micromorphological characters and illustrations were examined using dried voucher specimens under a light microscope, according to the methods described by Wu et al. (2022). Microscopic observations were carried out at magnifications of up to 1000× with a Nikon Eclipse 80i microscope (Nikon, Tokyo, Japan). Measurements and line drawings were prepared from slide mounts stained with Cotton Blue or Melzer’s reagent. Handmade sections of dried basidiomata were mounted in 5% KOH for 5 min and then treated with 2% Phloxine B (C_20_H_4_Br_4_Cl_2_K_2_O_5_). Basidiospore measurements were obtained from dried specimens. To better reflect size variability, the extreme 5% of values at both ends of the measurement range were excluded and are shown in parentheses. Color terminology follows the standards proposed by Petersen (1996).

### DNA extraction, PCR, and sequencing

Total genomic DNA was extracted from dried specimens using a CTAB rapid plant genome extraction kit (DN14; Aidlab Biotechnologies Co., Ltd., Beijing, China). PCR amplification was carried out following the manufacturer’s protocol, with minor modifications as described in previous studies (Liu et al. 2025).

The internal transcribed spacer (ITS) region was amplified using primers ITS4 (5'‐TCC TCC GCT TAT TGATAT GC‐3') and ITS5 (5'‐GGA AGT AAA AGT CGT AAC AAG G‐3') (White et al. 1990), and the nuclear large subunit rDNA (nLSU) region was amplified using LR0R (5'‐ACC CGC TGA ACT TAA GC‐3') and LR7 (5'‐TAC TAC CAC CAA GAT CT‐3') (https://sites.duke.edu/vilgalyslab/rdna_primers_for_fungi/). Amplification conditions for all loci followed the protocols described by Wang et al. (2024) and Zhao et al. (2025b). For the ITS region, initial denaturation was performed at 95 °C for 3 min, followed by 34 cycles of 94 °C for 40 s, 54 °C for 45 s and 72 °C for 1 min, with a final extension at 72 °C for 10 min; for the nLSU rDNA region, initial denaturation was performed at 94 °C for 1 min, followed by 34 cycles of 94 °C for 30 s, 50 °C for 1 min and 72 °C for 1.5 min, with a final extension at 72 °C for 10 min.

PCR products were purified and sequenced by the Beijing Genomics Institute (BGI), China, using the same primer pairs as those applied for amplification. All newly generated sequences were deposited in GenBank, and detailed sequence information is provided in Suppl. material 1.

### Phylogenetic relationships reconstruction

The ITS and nLSU sequences of *Resupinatus* and the outgroups *Hohenbuehelia flabelliformis* Phonemany & Raspé and *H. lageniformis* Phonemany & Raspé were used to reconstruct the phylogenetic trees. Each locus was aligned independently using MAFFT v7 (Katoh and Standley 2013), after which ambiguously aligned regions were excluded. The aligned ITS and nLSU datasets were concatenated in PhyloSuite v1.2.3 (Zhang et al. 2020) prior to phylogenetic reconstruction. The optimal nucleotide substitution model for the combined dataset was determined using ModelTest-NG v0.1.7 (Darriba et al. 2020).

Phylogenetic relationships were inferred using both maximum likelihood (ML) and Bayesian inference (BI) methods, implemented in RAxML v8 (Stamatakis 2014) and MrBayes v3.2.6 (Ronquist et al. 2012), respectively, following previously established procedures (Nie et al. 2025; Song et al. 2025; Wijesinghe et al. 2025). ML analyses were conducted with 1,000 bootstrap replicates under the selected model. BI was conducted for 2,000,000 generations with two independent runs, each consisting of four Markov chains (one cold and three heated). Trees were sampled every 1,000 generations, yielding a total of 2,000 trees. The first 25% of trees (500 trees) were discarded as burn-in, and the posterior distribution was estimated based on the remaining 1,500 trees. The burn-in fraction of 25% was determined by inspecting likelihood convergence and stationarity of parameter traces in MrBayes v3.2.6. Convergence between runs was further assessed by ensuring that the average standard deviation of split frequencies was below 0.01. Base frequencies were estimated during the analysis under the selected substitution model. Phylogenetic trees were visualized using FigTree v1.4.4. Nodes with ML bootstrap support ≥ 70% and BI posterior probabilities ≥ 0.95 were considered significantly supported.

## Results

### Phylogenetic analyses

**Figure 1.**
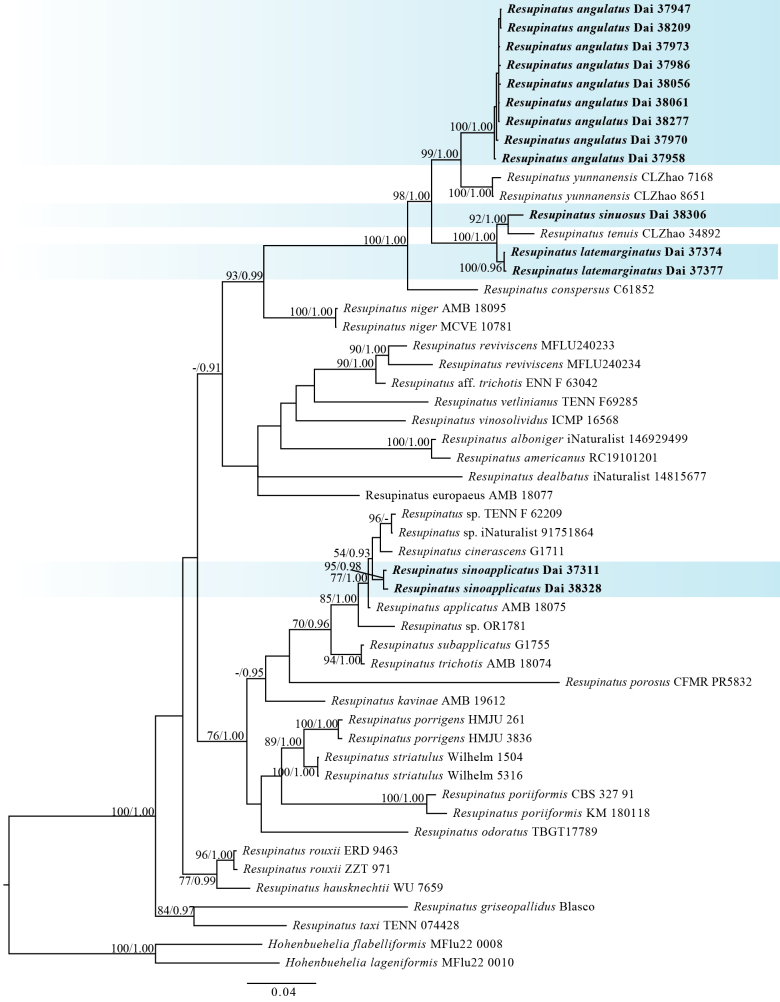
The Bayesian inference (BI) phylogeny of *Resupinatus* inferred from combined ITS and nLSU sequences, with *Hohenbuehelia flabelliformis* and *H. lageniformis* designated as outgroup taxa. Maximum likelihood (ML) bootstrap support values (BS ≥ 50%) and BI posterior probabilities (BPP ≥ 0.9) are indicated on the corresponding branches, while BS ≥ 70% and BPP ≥ 0.95 were considered significantly supported. The newly described species, *Resupinatus angulatus*, *R. latemarginatus*, *R. sinoapplicatus*, and *R. sinuosus*, are shown in bold. The scale bar (bottom) denotes the number of substitutions per site.

In this study, a total of 55 voucher specimens were used to construct the phylogenetic relationships, including 53 of *Resupinatus* and two of the outgroup *Hohenbuehelia*. A two-locus combined dataset (1,649 characters; ITS 1–746, nLSU 747–1,649) consisted of 1,082 constant, 139 parsimony-uninformative, and 428 parsimony-informative characters. Phylogenetic relationships were inferred using both ML and BI approaches based on the concatenated dataset, yielding largely congruent topologies. The GTRGAMMAIX and GTR + I + G substitution models were selected for the ML and BI analyses, respectively. The BI analysis recovered a tree topology essentially identical to that obtained from the ML analysis, with an average standard deviation of split frequencies of 0.007435, indicating satisfactory convergence. Given the high level of congruence between the two methods, only the BI tree is presented, with branch support values indicated as ML bootstrap support values (BS ≥ 50%) and BI posterior probabilities (BPP ≥ 0.9) in Fig. 1.

*Resupinatus angulatus* was recovered as the sister species to *R. yunnanensis* with good statistical support (98% BS and 1.00 BPP). *Resupinatus latemarginatus*, *R. sinuosus*, and *R. tenuis* are grouped together to form a distinct and well-supported clade (100% BS, 1.00 BPP). Another separate clade, comprising *Resupinatus sinoapplicatus*, *R. applicatus*, *R. cinerascens* (Cleland) Grgur., and three additional specimens, was also resolved with strong support (78% BS, 1.00 BPP).

### Taxonomy

#### Resupinatus angulatus

Taxon classificationFungiAgaricalesPleurotaceae

Q.X. Guan, H. Zhao, L. Zhuang, Yuan Yuan & Y.C. Dai sp. nov.

CE1F66FF-9908-56F8-AB88-F558268ED5E9

863200

Figs 2a, b, 3

##### Etymology.

*Angulatus* (Lat.): refers to the species having angular pores of hymenophore.

**Figure 2.**
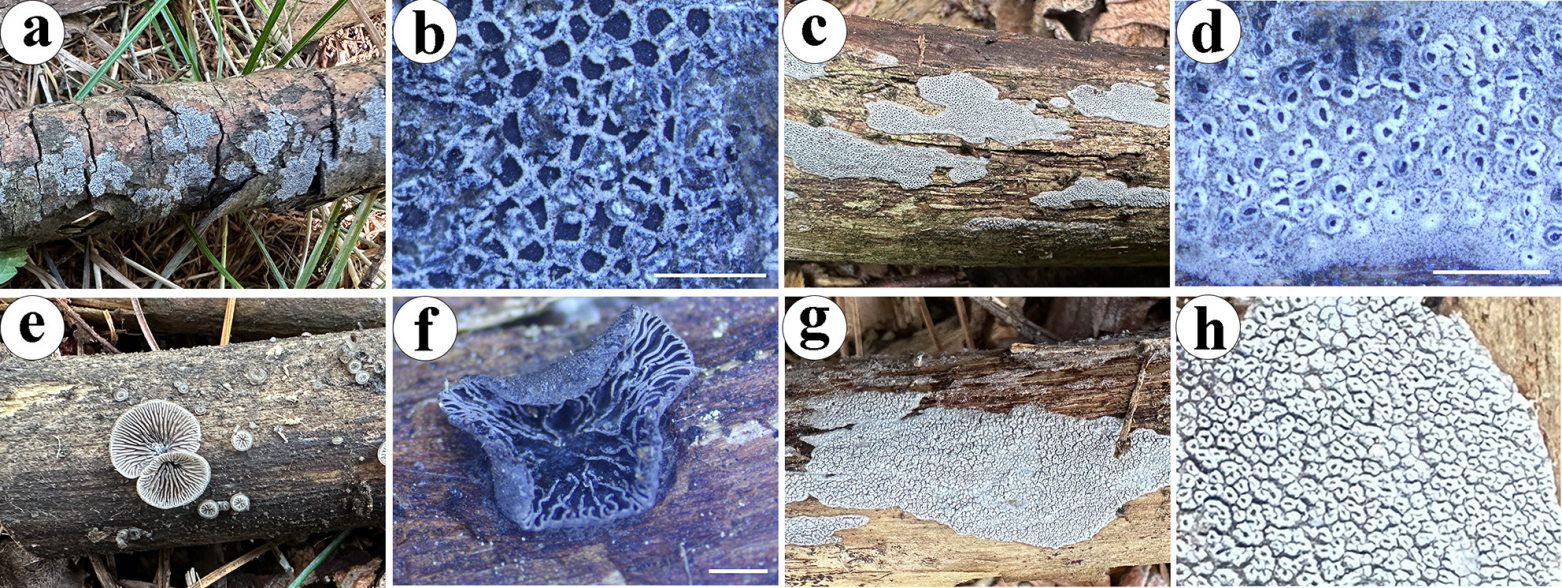
Basidiomata of *Resupinatus angulatus*, *R. latemarginatus*, *R. sinoapplicatus*, and *R. sinuosus*. **a, b**. *R. angulatus*, Dai 37973, holotype; **c, d**. *R. latemarginatus*, Dai 37377, holotype; **e, f**. *R. sinoapplicatus*, Dai 37311, holotype; **g, h**. *R. sinuosus*, 38306, holotype. Scale bars: 1 cm (**a, c, e, g**); 1 mm (**b, d, f, h**).

**Figure 3.**
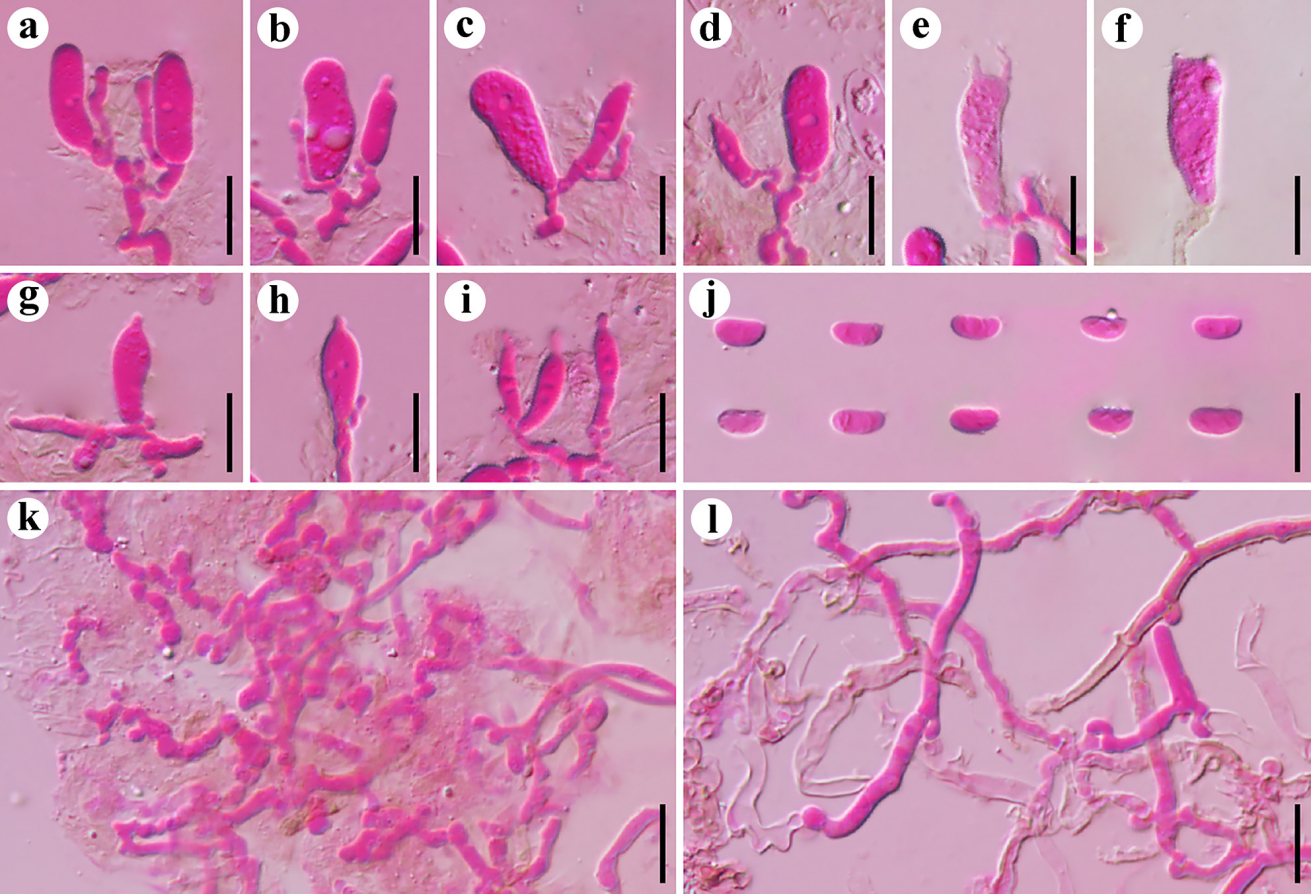
Microscopic structures of *Resupinatus angulatus* (holotype, Dai 37973). **a–f**. Basidia and basidioles; **g–i**. Cystidioles; **j**. Basidiospores; **k**. Generative hyphae; **l**. Subicular hyphae. Scale bars: 10 µm (**a–l**).

##### Holotype.

China • Jilin Province, Dunhua County, Yanminghu Forest Park, on fallen branch of *Larix olgensis*, collected by Yu-Cheng Dai, elev. 400 m, 43.641552°N, 128.527467°E, 13 September 2025, Dai 37973 (BJFC059232).

##### Description.

Basidiomata annual, resupinate, gelatinous, without odor or taste when fresh, becoming fragile upon drying, up to 1.5 cm long, 1 cm wide, 120 µm thick at center. Hymenial surface poroid, bluish gray when fresh, turning to dark bluish gray upon drying. Pores angular, 3–4 per mm, Sterile margin indistinct and slightly cream; subiculum felty, loosely attached to the substrate and densely packed, up to 0.2 mm thick.

Hyphal system monomitic; generative hyphae with clamp connections, colorless, thin-walled, frequently branched, flexuous, interwoven, 1–3 µm in diameter; subicular hyphae thin- to thick-walled, branched, interwoven, 1.5–3 µm in diameter, IKI–, CB–; tissues unchanged in KOH. Cystidia absent; cystidioles fusiform, colorless, thin-walled, 10–15 × 3–5.5 µm. Basidia short clavate to pyriform, with four sterigmata and a basal clamp connection, with a few guttules, 17–22 × 5.5–7 µm; basidioles similar to basidia but slightly smaller. Basidiospores curved cylindrical to allantoid, colorless, thin-walled, smooth, IKI–, CB–, (5.4–)5.6–7.3 × 2.6–3.6 µm, *L* = 6.51 µm, *W* = 3.05 µm, and *Q* = 2–2.28 (*n* = 120/4).

##### Habitat and distribution.

It is known from Heilongjiang and Jilin, Northeastern China, growing on fallen branches of *Larix olgensis* and *Pinus koraiensis*.

##### Additional specimens examined (paratypes).

China • Jilin Province, Dunhua County, Yanminghu Forest Park, collected by Yu-Cheng Dai, elev. 400 m, 43.641552°N, 128.527467°E, 13 September 2025: on fallen branch of *Pinus koraiensis*, Dai 37947; on fallen branch of *Larix olgensis*, Dai 37958 (BJFC059217), Dai 37970 (BJFC059229), and Dai 37986 (BJFC059245). • Heilongjiang Province: Tonghe County, Wulong National Park, on fallen branch of *Larix olgensis*, collected by Yu-Cheng Dai, elev. 29 m, 46.176944°N, 128.670833°E, 9 October 2025, Dai 38056 (BJFC059315) and Dai 38061 (BJFC059320); Fangzheng County, Longshan Forest Park, on fallen branch of *Larix olgensis*, collected by Yu-Cheng Dai, elev. 189 m, 45.822100°N, 129.394722°E, 11 October 2025, Dai 38209 (BJFC059468); Linkou County, Yuanyangfeng Forest Park, on fallen branch of *Larix olgensis*, collected by Yu-Cheng Dai, elev. 318 m, 45.594848°N, 129.553944°E, 11 October 2025, Dai 38277 (BJFC059536).

##### Notes.

*Resupinatus angulatus* is characterized by resupinate basidiomata; a poroid and bluish gray hymenial surface when fresh, dark bluish gray when drying; angular pores 3–4 per mm; a monomitic hyphal system bearing clamp connections; fusiform cystidioles; and curved cylindrical to allantoid basidiospores. *Resupinatus angulatus* is closely related to *R. yunnanensis* in the phylogeny (Fig. 1), but *R. yunnanensis* differs from *R. angulatus* by the absence of cystidioles and wider basidiospores (4.5–9 × 3.5–7 µm vs. 5.6–7.3 × 2.6–3.6 µm; Yang et al. 2023).

#### Resupinatus latemarginatus

Taxon classificationFungiAgaricalesPleurotaceae

Q.X. Guan, H. Zhao, L. Zhuang, Yuan Yuan & Y.C. Dai sp. nov.

E4731BD9-708A-55DC-A325-26BF4C0F6F1A

863202

Figs 2c, d, 4

##### Etymology.

*Latemarginatus* (Lat.): refers to the species having a wide sterile margin.

**Figure 4.**
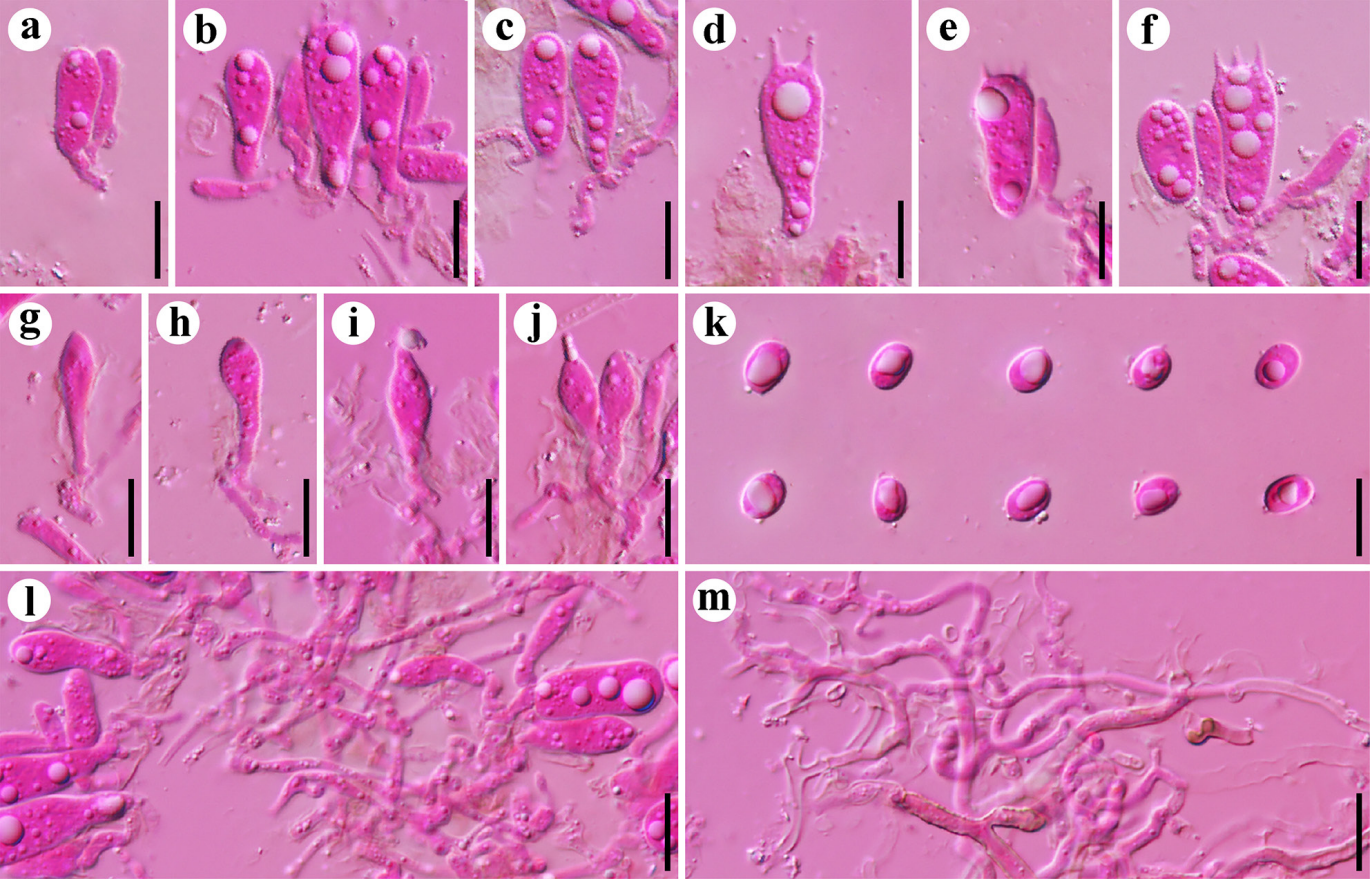
Microscopic structures of *Resupinatus latemarginatus* (holotype, Dai 37377). **a–f**. Basidia and basidioles; **g–j**. Cystidioles; **k**. Basidiospores; **l**. Generative hyphae; **m**. Subicular hyphae. Scale bars: 10 µm (**a–m**).

##### Holotype.

China • Heilongjiang Province, Wuchang County, Fenghuangshan Forest Park, on fallen branch of *Pinus koraiensis*, collected by Yu-Cheng Dai, elev. 786 m, 44.206724°N, 128.058065°E, 31 August 2025, Dai 37377 (BJFC058636).

##### Description.

Basidiomata annual, resupinate, gelatinous, without odor or taste when fresh, becoming fragile upon drying, up to 2 cm long, 0.6 cm wide, 100 µm thick at center. Hymenial surface poroid (isolated pores), ash-gray when fresh, turning to bluish gray upon drying. Pores circular to subcircular, 4–5 per mm; dissepiments covered in a dense mat of hairs. Sterile margin distinct, gray; subiculum felty, loosely attached to the substrate, and densely packed, up to 0.1 mm thick.

Hyphal system monomitic; generative hyphae with clamp connections, colorless, thin-walled, branched, interwoven, 1–2.5 µm in diameter; subicular hyphae thin- to slightly thick-walled, branched, interwoven, 1.5–3 µm in diameter, IKI–, CB–; tissues unchanged in KOH. Cystidia absent; cystidioles fusiform, colorless, thin-walled, 15–19 × 4.5–5.5 µm. Basidia short clavate to pyriform, with four sterigmata and a basal clamp connection, with a few guttules, 18–23 × 7–8.5 µm; basidioles similar to basidia but slightly smaller. Basidiospores ellipsoid, colorless, thin-walled, smooth, with one large guttule, IKI–, CB–, (5.7–)5.9–7.3 × (4.2–)4.4–5.4 µm, *L* = 6.59 µm, *W* = 4.85 µm, and *Q* = 1.34–1.37 (*n* = 60/2).

##### Habitat and distribution.

It is known from Heilongjiang, Northeastern China, growing on fallen branches of *Abies nephrolepis* and *Pinus koraiensis*.

##### Additional specimens examined (paratypes).

China • Heilongjiang Province, Wuchang County, Fenghuangshan Forest Park, on fallen branch of *Abies nephrolepis*, collected by Yu-Cheng Dai, elev. 786 m, 44.206724°N, 128.058065°E, 31 August 2025, Dai 37374 (BJFC058633).

##### Notes.

*Resupinatus latemarginatus* is characterized by resupinate basidiomata; a poroid and ash-gray hymenial surface when fresh, bluish gray when drying; circular to subcircular pores 4–5 per mm; a monomitic hyphal system bearing clamp connections; fusiform cystidioles; and ellipsoid basidiospores measuring 5.9–7.3 × 4.4–5.4 µm. *Resupinatus latemarginatus* is closely related to *R. sinuosus* and *R. tenuis* in the phylogeny (Fig. 1). However, *R. sinuosus* differs from *R. latemarginatus* by its sinuous to irregular pores, smaller basidia (16–18 × 7–8.5 µm vs. 18–23 × 7–8.5 µm) and basidiospores (5.3–6.6 × 4–5.2 µm vs. 5.9–7.3 × 4.4–5.4 µm), and *R. tenuis* is distinguished from *R. latemarginatus* by its smaller pores (7–10 per mm vs. 4–5 per mm), indistinct sterile margin, larger basidia (20.5–26.5 × 8.5–10 µm vs. 18–23 × 7–8.5 µm), and larger basidiospores (7.5–9 × 5–6 µm vs. 5.9–7.3 × 4.4–5.4 µm; Dong et al. 2025).

#### Resupinatus sinoapplicatus

Taxon classificationFungiAgaricalesPleurotaceae

Q.X. Guan, H. Zhao, L. Zhuang, Yuan Yuan & Y.C. Dai sp. nov.

45FFA4E5-A895-57AB-9DA8-C37DF23C68A1

863203

Figs 2e, f, 5

##### Etymology.

*Sinoapplicatus* (Lat.): refers to the species similar to *Resupinatus applicatus*, but occurring in China.

**Figure 5.**
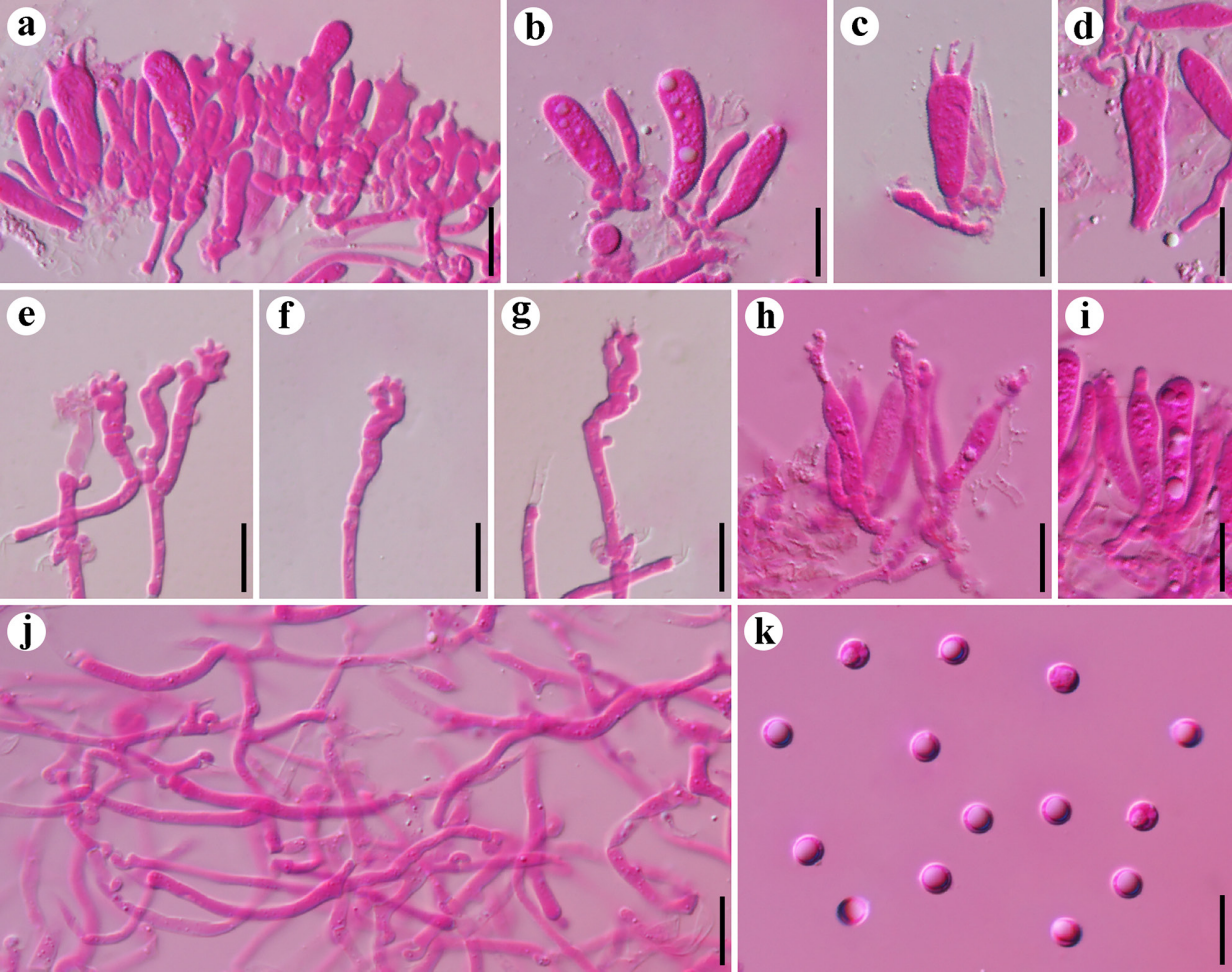
Microscopic structures of *Resupinatus sinoapplicatus* (holotype, Dai 37311). **a**. A section of hymenium; **b–d**. Basidia and basidioles; **e–g**. Cheilocystidia; **h, i**. Pleurocystidia; **j**. Generative hyphae; **k**. Basidiospores. Scale bars: 10 µm (**a–k**).

##### Holotype.

China • Heilongjiang Province, Wuchang County, Chenxiangdian Forest Farm, on fallen branch of *Pinus koraiensis*, collected by Yu-Cheng Dai, elev. 212 m, 44.957241°N, 127.727710°E, 30 August 2025, Dai 37311 (BJFC058570).

##### Description.

Basidiomata annual, sessile, gelatinous, flabelliform or occasional suborbicular, laterally or dorsally attached to substrate, fragile and watery when fresh, becoming firm-gelatinous when dry. Pileus up to 10 mm in diam., orbicular when juvenile, becoming flabelliform when mature; upper surface pale mouse gray when fresh, becoming blackish blue when dry, smooth, sometimes pruinose; margin unrolled to incurved when juvenile, becoming straight, entire. Hymenium lamellate, lamellae with different lengths. Stipe absent. Context dark brown, leathery when dry, up to 1 mm thick.

Hyphal system monomitic; generative hyphae with clamp connections, colorless, thin-walled, branched, interwoven, 1–2.5 µm in diameter. Cheilocystidia clavate to coralloid with irregular, bifurcate, multifurcate, or rarely nodulose, thin-walled, hyaline, 8–15 × 5–7 µm, arising from generative hyphae. Pleurocystidia present, fusiform to with branched apices, 20–25 × 3.5–5 µm. Basidia clavate, with four sterigmata and a basal clamp connection, with a few guttules, 17–23 × 6–7.5 µm; basidioles similar to basidia but slightly smaller. Basidiospores globose, colorless, thin-walled, smooth, with one large guttule, IKI–, CB–, (3.8–)3.9–5 × (3.6–)3.7–4.7 µm, *L* = 4.4 µm, *W* = 4.1 µm, and *Q* = 1.05–1.07 (*n* = 60/2).

##### Habitat and distribution.

It is known from Heilongjiang, Northeastern China, growing on fallen branches of *Pinus koraiensis*.

##### Additional specimens examined (paratypes).

China • Heilongjiang Province, Fangzheng County, Shuangzishan Forest Park, on fallen branch of *Pinus koraiensis*, collected by Yu-Cheng Dai, elev. 187 m, 45.677502°N, 129.001192°E, 12 October 2025, Dai 38328 (BJFC059587).

##### Notes.

*Resupinatus sinoapplicatus* is characterized by sessile, flabelliform or occasionally suborbicular basidiomata; pale mouse-gray pileus when fresh, blackish blue when drying; lamellate hymenium; a monomitic hyphal system bearing clamp connections; clavate to coralloid cheilocystidia; fusiform pleurocystidia with branched apices; and globose basidiospores. In the phylogeny (Fig. 1), *R. sinoapplicatus* is closely allied with *R. applicatus* and *R. cinerascens*. However, *R. applicatus* is separable from *R. sinoapplicatus* by its subglobose to globose basidiospores (*Q* = 1.07–1.15 vs. 1.05–1.07), narrower basidia (19–25 × 5–6.1 µm vs. 17–23 × 6–7.5 µm), and fusiform to cylindrical-clavate cheilocystidia (Gonou-Zagou et al. 2011); *R. cinerascens* differs from *R. sinoapplicatus* by its larger basidiospores (6 × 3.5 µm vs. 4.39 × 4.13 µm; Cleland 1927).

#### Resupinatus sinuosus

Taxon classificationFungiAgaricalesPleurotaceae

Q.X. Guan, H. Zhao, L. Zhuang, Yuan Yuan & Y.C. Dai sp. nov.

AA35EEAA-1D62-5551-9F49-F732372088EC

863204

Figs 2g, h, 6

##### Etymology.

*Sinuosus* (Lat.): refers to the species having sinuous pores.

**Figure 6.**
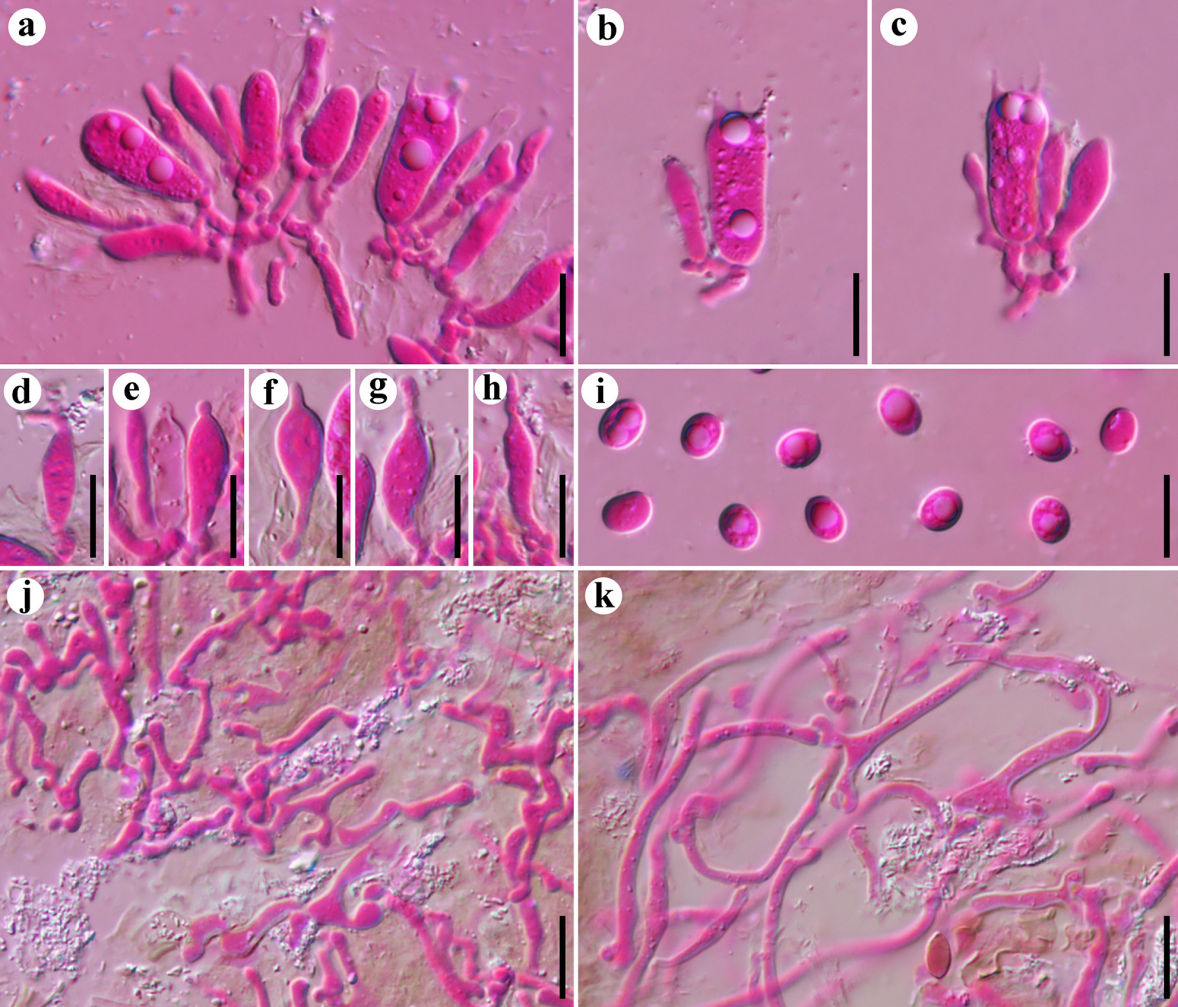
Microscopic structures of *Resupinatus sinuosus* (holotype, Dai 38306). **a**. A section of hymenium; **b, c**. Basidia; **d–h**. Cystidioles; **i**. Basidiospores; **j**. Generative hyphae; **k**. Subicular hyphae. Scale bars: 10 µm (**a–k**).

##### Holotype.

China • Heilongjiang Province, Linkou County, Sandaotong Virgin Forest, on rotten wood of *Abies nephrolepis*, collected by Yu-Cheng Dai, elev. 451 m, 45.725278°N, 129.459167°E, 11 October 2025, Dai 38306 (BJFC059565).

##### Description.

Basidiomata annual, resupinate, gelatinous, without odor or taste when fresh, becoming fragile upon drying, up to 8 cm long, 1 cm wide, 100 µm thick at center. Hymenial surface poroid (isolated pores), ash-gray when fresh and dry. Pores sinuous to irregular, 3–4 per mm; dissepiments covered in a dense mat of hairs. Sterile margin distinct, gray; subiculum felty, loosely attached to the substrate, and densely packed, up to 0.1 mm thick.

Hyphal system monomitic; generative hyphae with clamp connections, colorless, thin-walled, frequently branched, flexuous, interwoven, 1–3 µm in diameter; subicular hyphae thin- to slightly thick-walled, branched, interwoven, straight, 1–3.5 µm in diameter, IKI–, CB–; tissues unchanged in KOH. Cystidia absent; cystidioles fusiform, sometimes with branched apices, colorless, thin-walled, 17–22 × 3.5–6.5 µm. Basidia short clavate, with four sterigmata and a basal clamp connection, with a few guttules, 16–18 × 7–8.5 µm; basidioles similar to basidia but slightly smaller. Basidiospores ellipsoid, colorless, thin-walled, smooth, with one or two guttules, IKI–, CB–, (5–)5.3–6.6 × (3.9–)4–5.2 µm, *L* = 5.93 µm, *W* = 4.57 µm, and *Q* = 1.29 (*n* = 30/1).

##### Habitat and distribution.

It is known from Heilongjiang, Northeastern China, growing on rotten wood of *Abies nephrolepis*.

##### Notes.

*Resupinatus sinuosus* is characterized by resupinate, gelatinous basidiomata; a poroid and ash-gray hymenial surface; sinuous to irregular pores 3–4 per mm; a monomitic hyphal system bearing clamp connections; fusiform cystidioles; and ellipsoid basidiospores. In the phylogeny (Fig. 1), *R. sinuosus* is closely related to *R. latemarginatus* and *R. tenuis*, but *R. tenuis* is distinguished from *R. sinuosus* by smaller pores (7–10 per mm vs. 3–4 per mm), larger basidia (20.5–26.5 × 8.5–10 µm vs. 16–18 × 7–8.5 µm), and larger basidiospores (7.5–9 × 5–6 µm vs. 5.3–6.6 × 4–5.2 µm; Dong et al. 2025). For differences between *R. latemarginatus* and *R. sinuosus*, see notes under *R. latemarginatus*.

## Discussion

In this study, four new species (*Resupinatus angulatus*, *R. latemarginatus*, *R. sinoapplicatus*, and *R. sinuosus*) are described from coniferous wood (*Abies nephrolepis*, *Larix olgensis*, and *Pinus koraiensis*) in Jilin and Heilongjiang provinces, Northeast China, based on phylogenetic evidence and morphological characteristics.

For a long time, the taxonomic placement of *Resupinatus* has undergone repeated revisions, which hindered a clear understanding of its species diversity. In recent years, however, *Resupinatus* has increasingly been recognized as representing an independent family, *Resupinataceae*, within the *Agaricales* (Hyde et al. 2024; Vizzini et al. 2024). Numerous species have been described from subtropical to tropical forests (Yang et al. 2023; Vizzini et al. 2024; Dong et al. 2025), and a few species have also been found in coniferous forests or on bamboos (Carpouron et al. 2024; Liu et al. 2024).

Temperate-boreal coniferous trees are among the most important components of forest ecosystems in the Northern Hemisphere, providing crucial ecological niches for macrofungi, including wood-decaying fungi (Palla et al. 2023). In Northeast China, *Abies nephrolepis*, *Larix olgensis*, and *Pinus koraiensis* are dominant coniferous tree species. Although polypores on these hosts have been studied (Dai 2000; Dai and Penttilä 2006; Dai et al. 2007), the diversity of other wood-decaying fungi associated with these hosts has long remained insufficiently explored. A previous study has reported 174 wood-inhabiting fungal species on *Pinus koraiensis* (Zhou et al. 2024), and this number is expected to increase with more extensive sampling.

### Key to known species of *Resupinatus* in China

**Table:** 

1	Basidiomata sessile, with lamellate hymenophore	**2**
–	Basidiomata resupinate, with poroid or cyphelloid hymenophore	**6**
2	Pileus surface pale mouse gray	***R. sinoapplicatus***
–	Pileus surface red-brown, greyish brown to black brown or black	**3**
3	Pileus surface red-brown toward the margin	***R. porrigens***
–	Pileus surface greyish brown to black brown or black	**4**
4	Basidiospores oblong ellipsoid, 5.5–6.6 × 3–4 µm	***R. alboniger***
–	Basidiospores globose to subglobose	**5**
5	Pileal surface yellow to brown tomentose; cystidioles > 20 µm in length	***R. applicatus***
–	Pileal surface black tomentose; cystidioles < 20 µm in length	***R. trichotis***
6	Basidiomata with angular or sinuous to irregular pores	**7**
–	Basidiomata with circular to subcircular pores	**8**
7	Cystidioles 17–22 × 3.5–6.5 µm; basidiospores ellipsoid, 5.3–6.6 × 4–5.2 µm	***R. sinuosus***
–	Cystidioles 10–15 × 3–5.5 µm; basidiospores allantoid, 5.6–7.3 × 2.6–3.6 µm	***R. angulatus***
8	Pores smaller, > 7 per mm; basidia > 8.5 µm in width	***R. tenuis***
–	Pores larger, < 7 per mm; basidia < 8.5 µm in width	**9**
9	Cystidioles absent; crystal-encrusted branched hyphae present	***R. yunnanensis***
–	Cystidioles present; crystal-encrusted branched hyphae absent	***R. latemarginatus***

## Supplementary Material

XML Treatment for Resupinatus angulatus

XML Treatment for Resupinatus latemarginatus

XML Treatment for Resupinatus sinoapplicatus

XML Treatment for Resupinatus sinuosus

## Additional information

### Artificial Intelligence (AI) use

The authors accept full responsibility for the content of the manuscript, including the disclosure of any use of AI.

No AI tools were used in the preparation of this manuscript.

### Author ORCIDs

L. Zhuang https://orcid.org/0009-0006-5215-0192

Q.-X. Guan https://orcid.org/0000-0002-7072-080X

Y.-C. Dai https://orcid.org/0000-0002-6523-0320

Y. Yuan https://orcid.org/0000-0001-6674-9848

H. Zhao https://orcid.org/0000-0003-2938-5613

### Data availability

The newly generated sequences have been uploaded to GenBank (Suppl. material 1).

## Supplementary materials

Supplementary material 1 GenBank accession numbers Data type docx Explanation note GenBank accession numbers of sequences used in this study. – refers to unavailable data. “” refers to species in publishing. New species are shown in bold. https://binary.pensoft.net/file/1645793This dataset is made available under the Open Database License (http://opendatacommons.org/licenses/odbl/1.0/). The Open Database License (ODbL) is a license agreement intended to allow users to freely share, modify, and use this Dataset while maintaining this same freedom for others, provided that the original source and author(s) are credited. Lei Zhuang, Qian-Xin Guan, Yu-Cheng Dai, Yuan Yuan, Heng Zhao
