# Supplementary material for: Four new species of Resupinatus (Agaricales, Basidiomycota) on coniferous trees in Northeast China
Source: MycoKeys. 2026 May 15;132:173–89. doi: 10.3897/mycokeys.132.188107 (PMC13197816; doi:10.3897/mycokeys.132.188107)
Supplement: Supplementary material 1 — GenBank accession numbers [file mycokeys-132-173-s001.docx]

**Table S1.** The GenBank accession numbers of sequences used in this study. – refers to the unavailability of the data. “”refers to the species in publishing. New species are shown in bold.

| Species name | Voucher No. | GenBank Accession no. | |
| --- | --- | --- | --- |
|  |  | ITS | nLSU |
| *Hohenbuehelia flabelliformis* | MFlu22 0008 | OP236779 | OM521957 |
| *Hohenbuehelia lageniformis* | MFlu22 0010 | OP236781 | OM521958 |
| *Resupinatus abieticola* | ATHUM 10407 | PX057674 | PX057680 |
| *Resupinatus abieticola* | ATHUM 10408 | PX057675 | PX057681 |
| *Resupinatus abieticola* | ATHUM 10409 | PX057676 | PX057682 |
| *Resupinatus alboniger* | iNaturalist 146929499 | OR081334 | – |
| *Resupinatus americanus* | RC19101201 | OM397444 | – |
| ***Resupinatus angulatus*** | **Dai 37947** | **PX982925** | **PX982939** |
| ***Resupinatus angulatus*** | **Dai 37958** | **PX982926** | **PX982940** |
| ***Resupinatus angulatus*** | **Dai 37970** | **PX982927** | **PX982941** |
| ***Resupinatus angulatus*** | **Dai 37973** | **PX982928** | **PX982942** |
| ***Resupinatus angulatus*** | **Dai 37986** | **PX982929** | **PX982943** |
| ***Resupinatus angulatus*** | **Dai 38056** | **PX982930** | **PX982944** |
| ***Resupinatus angulatus*** | **Dai 38061** | **PX982931** | **PX982945** |
| ***Resupinatus angulatus*** | **Dai 38209** | **PX982932** | **PX982946** |
| ***Resupinatus angulatus*** | **Dai 38277** | **PX982933** | **PX982947** |
| *Resupinatus applicatus* | AMB 18075 | KU355368 | KU355411 |
| *Resupinatus cinerascens* | G1711 | – | MK278551 |
| *Resupinatus conspersus* | C61852 | AY571061 | AY571024 |
| *Resupinatus dealbatus* | iNaturalist 14815677 | ON416908 | – |
| *Resupinatus europaeus* | AMB 18077 | KU355366 | KU355410 |
| *Resupinatus griseopallidus* | Blasco | MG553642 | MG553649 |
| *Resupinatus hausknechtii* | WU 7659 | KU355370 | KU355412 |
| *Resupinatus kavinae* | AMB 19612 | MG553643 | MG553650 |
| ***Resupinatus latemarginatus*** | **Dai 37374** | **PX982934** | **PX982948** |
| ***Resupinatus latemarginatus*** | **Dai 37377** | **PX982935** | **PX982949** |
| *Resupinatus niger* | AMB 18095 | KU355371 | KU355413 |
| *Resupinatus niger* | MCVE 10781 | KU355372 | KU355414 |
| *Resupinatus odoratus* | TBGT17789 | MT452498 | – |
| *Resupinatus porosus* | CFMR PR5832 | NR_119556 | – |
| *Resupinatus poriiformis* | CBS 327.91 | AY571062 | AY571025 |
| *Resupinatus poriiformis* | KM 180118 | MZ159469 | – |
| *Resupinatus porrigens* | HMJU 261 | NR_198332 | NG_243157 |
| *Resupinatus* *porrigens* | HMJU 3826 | OP729420 | OP729419 |
| *Resupinatus reviviscens* | MFLU240233 | PQ036937 | PQ036942 |
| *Resupinatus reviviscens* | MFLU240234 | PQ036938 | PQ036943 |
| *Resupinatus rouxii* | ERD 9463 | OP289290 | – |
| *Resupinatus rouxii* | ZZT 971 | MH168326 | MH190787 |
| ***Resupinatus sinoapplicatus*** | **Dai 37311** | **PX982936** | **PX982950** |
| ***Resupinatus sinoapplicatus*** | **Dai 37328** | **PX982937** | – |
| ***Resupinatus sinuosus*** | **Dai 38306** | **PX982938** | **PX982951** |
| *Resupinatus* sp. | iNaturalist 91751864 | OQ389423 | – |
| *Resupinatus* sp. | OR1781 | PQ475810 | PQ036943 |
| *Resupinatus* sp. | TENN F 62209 | KP026229 | – |
| *Resupinatus striatulus* | Wilhelm 1504 | KU355374 | – |
| *Resupinatus striatulus* | Wilhelm 5316 | MH137831 | MH169342 |
| *Resupinatus subapplicatus* | G1755 | – | MK278557 |
| *Resupinatus taxi* | TENN 074428 | MH558280 | – |
| *Resupinatus tenuis* | CLZhao 34892 | PV197932 | PV197946 |
| *Resupinatus trichotis* | AMB 18074 | KU355378 | KU355416 |
| *Resupinatus vetlinianus* | TENN F69285 | KP026243 | KP987309 |
| *Resupinatus vinosolividus* | ICMP 16568 | MZ325958 | – |
| *Resupinatus* aff*. trichotis* | ENN F 63042 | KP026231 | – |
| *Resupinatus yunnanensis* | CLZhao 7168 | OP901838 | – |
| *Resupinatus yunnanensis* | CLZhao 8651 | OP901839 | OP904197 |
